# Artificial intelligence in health insurance fraud detection: a global and regional systematic review

**DOI:** 10.3389/fpubh.2026.1863242

**Published:** 2026-10-15

**Authors:** Naof Faiz Saleem Al-Ansary

**Affiliations:** Department of Public Health, College of Public Health, Imam Abdulrahman bin Faisal University, Dammam, Saudi Arabia

**Keywords:** artificial intelligence, deep learning, health insurance fraud, machine learning, PRISMA 2020, systematic review

## Abstract

Health insurance fraud imposes substantial financial burdens on healthcare systems and reduces the efficiency of healthcare resource utilization worldwide. Traditional fraud detection approaches often struggle to analyse large and complex healthcare claims data, making artificial intelligence (AI) an increasingly important tool for identifying fraudulent activities. This study systematically reviews global and regional evidence on AI applications in health insurance fraud detection, with particular emphasis on methodological approaches, performance evaluation, and implementation challenges. This systematic review followed the PRISMA 2020 guidelines. Seven electronic databases were searched for studies published between 2016 and 2025. Titles and abstracts were screened using the Silvi AI platform, followed by eligibility assessment according to pre-defined inclusion and exclusion criteria. Data were extracted using a standardized framework, and methodological quality was assessed using the Drummond 10-item checklist. From 450 identified records, 33 studies met the eligibility criteria. The included studies demonstrated substantial heterogeneity in datasets, AI methodologies, fraud types, and performance evaluation metrics. Machine learning was the most frequently applied approach, followed by deep learning, graph-based learning, and hybrid AI frameworks. Although AI-based methods consistently improved fraud detection performance, challenges related to dataset imbalance, standardized reporting, interpretability, external validation, and economic evaluation remain significant barriers to widespread implementation. AI demonstrates considerable potential for improving health insurance fraud detection. However, future research should prioritize standardized evaluation frameworks, explainable AI, external validation across diverse healthcare systems, and economic assessment to support the responsible and scalable deployment of AI-enabled fraud detection systems.

## 1 Introduction

Health insurance fraud is a persistent global challenge that imposes substantial financial burdens on healthcare systems, diverts limited healthcare resources, and undermines public trust in insurance programs. The National HealthCare Anti-Fraud Association (1) estimates that healthcare fraud results in billions of dollars in annual losses worldwide. Common fraudulent practices include upcoding, phantom billing, duplicate claims, unnecessary medical procedures, identity misuse, prescription fraud, and collusive arrangements among healthcare providers, beneficiaries, insurers, and other stakeholders (2, 3). As healthcare reimbursement systems become increasingly digital and data-intensive, traditional fraud detection approaches based on manual auditing and rule-based systems have become less effective in identifying sophisticated and evolving fraudulent behaviors.

Recent advances in artificial intelligence (AI) have transformed health insurance fraud detection by enabling the analysis of large-scale claims data and the identification of complex fraud patterns that are difficult to detect using conventional statistical approaches. Machine learning, deep learning, graph neural networks, anomaly detection techniques, and explainable artificial intelligence (XAI) have demonstrated promising performance in detecting fraudulent claims, provider collusion, and abnormal healthcare utilization (4–7). Supervised learning methods, including logistic regression, decision trees, random forests, support vector machines, gradient boosting algorithms, and neural networks, have been widely adopted for fraud classification, whereas unsupervised and semi-supervised approaches are frequently employed to identify anomalous claim patterns when labeled fraud data are limited (8, 9).

Regardless of the learning paradigm, one of the most significant challenges in health insurance fraud detection is the severe class imbalance inherent in healthcare claims datasets, where fraudulent claims typically represent only a very small proportion of the total observations. This imbalance affects the performance of both supervised and unsupervised AI models and may result in misleadingly high accuracy while failing to identify minority fraud cases. To address this challenge, researchers have proposed a variety of data-level and algorithm-level solutions, including random oversampling, under-sampling, the Synthetic Minority Over-sampling Technique (SMOTE) (48), SMOTE-ENN (Edited Nearest Neighbors), cost-sensitive learning, ensemble learning, graph-based learning, and hybrid deep learning frameworks (10–13). More recently, hybrid AI models have integrated graph neural networks, deep learning, explainable AI, and generative adversarial networks (GANs) to improve fraud detection performance while enhancing model robustness and interpretability. For example, Mao et al. (14) proposed a GAN–XGBoost architecture for medical insurance fraud detection, whereas Villegas-Ortega et al. (15) demonstrated the effectiveness of supervised machine learning for identifying suspicious health insurance claims. Similarly, Prova (49) highlighted the growing application of machine learning techniques in healthcare fraud detection, reflecting the rapidly evolving nature of AI-enabled fraud detection research.

Health insurance fraud encompasses multiple stakeholders and fraud types rather than a single form of fraudulent activity. Fraud may be committed by healthcare providers through false billing, upcoding, unnecessary procedures, phantom claims, or provider collusion; by beneficiaries through identity misuse and fraudulent reimbursement claims; or through organized collusive networks involving multiple actors within healthcare reimbursement systems. Accordingly, this systematic review adopts a comprehensive perspective by examining AI-based approaches developed to detect fraud across healthcare providers, beneficiaries, insurers, and other entities involved in health insurance systems. This broader scope enables comparison of methodological approaches, datasets, AI techniques, evaluation metrics, and implementation challenges across diverse healthcare environments while identifying common methodological trends and research gaps.

Despite the rapid growth of AI-based health insurance fraud detection research, the available evidence remains fragmented across different healthcare systems, fraud types, datasets, AI methodologies, and evaluation practices. Considerable heterogeneity exists in the reported performance metrics, methodological quality, implementation contexts, and validation strategies, making direct comparisons across studies challenging. Furthermore, relatively limited attention has been devoted to issues such as standardized reporting, dataset imbalance, model interpretability, fairness, economic evaluation, governance, and the practical deployment of AI-based fraud detection systems. Fairness refers to the ability of the AI models to make predictions fairly without introducing systematic biases toward certain groups of patients, toward specific healthcare providers, toward any population (16). While Governance is the policies, frameworks, ethical rules, accountability frameworks, and mechanisms that are in place to ensure the responsible creation, deployment, and operation of AI systems (17). There are aspects of fairness and governance that are related to model interpretability but are broader in scope to cover more of trustworthy AI. Interpretability is about understanding the models' predictions, fairness is about fair decision making and governance is about the organization and regulation of the responsible use of AI (18).

Consequently, a comprehensive and critical synthesis of the current evidence is needed to evaluate methodological developments, identify knowledge gaps, and provide recommendations for future research and practical implementation. In response to these needs, the present study conducts a PRISMA-guided global and regional systematic review of AI applications in health insurance fraud detection, with particular emphasis on AI techniques, methodological quality, performance evaluation, implementation challenges, and future research directions.

### 1.1 Objectives

This systematic review aims to (i) identify and categorize the artificial intelligence techniques applied to health insurance fraud detection; (ii) examine the characteristics of the datasets, fraud types, and performance evaluation metrics reported in the literature; (iii) assess the methodological quality of the included studies; (iv) compare global and regional research trends and implementation approaches; (v) identify methodological, technical, and practical research gaps; and (vi) provide evidence-based recommendations to guide future research and the responsible implementation of AI-enabled health insurance fraud detection systems.

### 1.2 Research questions and conceptual framework

This systematic review is guided by the following research questions:

**RQ1:** What artificial intelligence techniques have been applied to detect health insurance fraud, and how have these approaches evolved across different healthcare settings?

**RQ2:** What types of health insurance fraud, datasets, and healthcare stakeholders (e.g., providers, beneficiaries, and insurers) have been investigated in the existing literature?

**RQ3:** Which performance evaluation metrics (e.g., Accuracy, Precision, Recall, F1-score, AUROC, and PR-AUC) are most frequently used to assess AI-based health insurance fraud detection models, and what methodological trends can be identified?

### 1.3 Economic dimension

**RQ4:** What evidence exists regarding the economic implications of AI-based health insurance fraud detection, including cost-effectiveness, operational efficiency, resource utilization, and financial sustainability?

### 1.4 Implementation dimension

**RQ5:** What technical, organizational, ethical, and regulatory challenges influence the implementation of AI-enabled health insurance fraud detection systems, and what research gaps and future directions are identified in the existing literature?

## 2 Research methodology

### 2.1 Methods

The present systematic review was conducted in accordance with the *Cochrane Handbook for Systematic Reviews of Interventions* (46) and followed the *Preferred Reporting Items for Systematic Reviews and Meta-Analyses (PRISMA 2020)* guidelines and checklist (19, 20).

### 2.2 Search strategy

A comprehensive literature search was conducted across seven electronic databases, namely Scopus, Web of Science, OVID Medline, Embase, PubMed, Dimensions, and OpenAlex, to ensure broad coverage of interdisciplinary research on AI-based health insurance fraud detection. The detailed search strategies for each database are presented in Table 1.

**Table 1:** Search strategy.

Database	Search strategy	Number of records
Scopus	TITLE-ABS-KEY ((“artificial intelligence” OR AI OR “machine learning” OR ML OR “deep learning” OR DL OR “neural network^*^” OR “predictive analytics” OR “data mining” OR “natural language processing” OR NLP OR “expert system^*^”) AND (“insurance fraud” OR “fraud detection” OR “fraudulent claim^*^” OR “fraud analytics” OR “fraud management”) AND (“health insurance” OR “medical insurance” OR “Cooperative Health Insurance” OR CHI OR “healthcare financing” OR “claims data” OR “Universal Insurance”)) AND PUBYEAR > 2015 AND PUBYEAR < 2026 AND (LIMIT-TO (DOCTYPE, “ar”)) AND (LIMIT-TO (LANGUAGE, “English”))	91
Web of science	(TS=((“artificial intelligence” OR AI OR “machine learning” OR ML OR “deep learning” OR DL OR “neural network^*^” OR “predictive analytics” OR “data mining” OR “natural language processing” OR NLP OR “expert system^*^”) AND (“insurance fraud” OR “fraud detection” OR “fraudulent claim^*^” OR “fraud analytics” OR “fraud management”) AND (“health insurance” OR “medical insurance” OR “Cooperative Health Insurance” OR CHI OR “healthcare financing” OR “claims data” OR “Universal Insurance”)))	53
Medline		15
Embase	(‘artificial intelligence'/exp OR ai:ti,ab,kw OR ‘machine learning'/exp OR ml:ti,ab,kw OR ‘deep learning'/exp OR dl:ti,ab,kw OR ‘neural network^*^':ti,ab,kw OR ‘predictive analytics':ti,ab,kw OR ‘data mining'/exp OR ‘natural language processing'/exp OR nlp:ti,ab,kw OR ‘expert system^*^':ti,ab,kw) AND (‘insurance fraud':ti,ab,kw OR ‘fraud detection':ti,ab,kw OR ‘fraudulent claim^*^':ti,ab,kw OR ‘fraud management':ti,ab,kw OR ‘fraud analytics':ti,ab,kw) AND (‘health insurance'/exp OR ‘medical insurance':ti,ab,kw OR ‘cooperative health insurance':ti,ab,kw OR chi:ti,ab,kw OR ‘healthcare financing':ti,ab,kw OR ‘claims data':ti,ab,kw OR ‘universal insurance':ti,ab,kw)	15
PubMed	((“Artificial Intelligence”[Mesh] OR “artificial intelligence”[tiab] OR AI[tiab] OR “Machine Learning”[Mesh] OR “machine learning”[tiab] OR ML[tiab] OR “Deep Learning”[tiab] OR DL[tiab] OR “Neural Networks, Computer”[Mesh] OR “neural network^*^”[tiab] OR “predictive analytics”[tiab] OR “Data Mining”[Mesh] OR “data mining”[tiab] OR “Natural Language Processing”[Mesh] OR NLP[tiab] OR “expert system^*^”[tiab]) AND (“Insurance, Health”[Mesh] OR “health insurance”[tiab] OR “medical insurance”[tiab] OR “Cooperative Health Insurance”[tiab] OR CHI[tiab] OR “claims data”[tiab]) AND (“Fraud”[Mesh] OR “insurance fraud”[tiab] OR “fraud detection”[tiab] OR “fraudulent claim^*^”[tiab] OR “fraud management”[tiab] OR “fraud analytics”[tiab] OR “Universal Insurance”[tiab])	16
Dimensions	(“artificial intelligence” OR AI OR “machine learning” OR ML OR “deep learning” OR DL OR “neural network^*^” OR “predictive analytics” OR “data mining” OR “natural language processing” OR NLP OR “expert system^*^”) AND (“insurance fraud” OR “fraud detection” OR “fraudulent claim^*^” OR “fraud analytics” OR “fraud management”) AND (“health insurance” OR “medical insurance” OR “Cooperative Health Insurance” OR CHI OR “healthcare financing” OR “claims data” OR “Universal Insurance”)	138
OpenAlex		122

### 2.3 Inclusion/exclusion criteria

The included studies focused on AI applications in health insurance fraud detection, reporting outcomes such as effectiveness, efficiency, scalability, economic viability, and implementation challenges. Eligible studies were quantitative or computational investigations published in English between January 2016 and March 2025. Studies were excluded if they did not address health insurance fraud, lacked relevant outcome measures, or were systematic reviews, editorials, conference abstracts, commentaries, opinion papers, duplicate records, retracted publications, or articles with inaccessible full texts.

To ensure methodological consistency and feasibility, the review was limited to peer-reviewed English-language publications. English was selected because it represents the dominant language of international scientific communication, enabling consistent screening, interpretation, and data extraction while minimizing potential translation bias. The selected time frame captures the rapid advancement of contemporary AI approaches including machine learning, deep learning, graph neural networks, and explainable AI which have become increasingly prominent in health insurance fraud detection since 2016. Earlier studies primarily relied on traditional statistical and rule-based methods and were therefore considered less representative of current AI-driven methodologies.

### 2.4 Data extraction

Comprehensive data extraction was conducted using standardized Microsoft Excel worksheets. Information extracted from each eligible study included article characteristics (authors, publication year, journal, and title), country of study, data source, AI techniques, performance evaluation metrics, fraud type, reported outcomes, key findings, funding information, and study limitations (Figure 1). A standardized extraction framework was developed to ensure consistency across all included studies and to facilitate comparative synthesis of methodological characteristics and reported outcomes.

**Figure 1:**
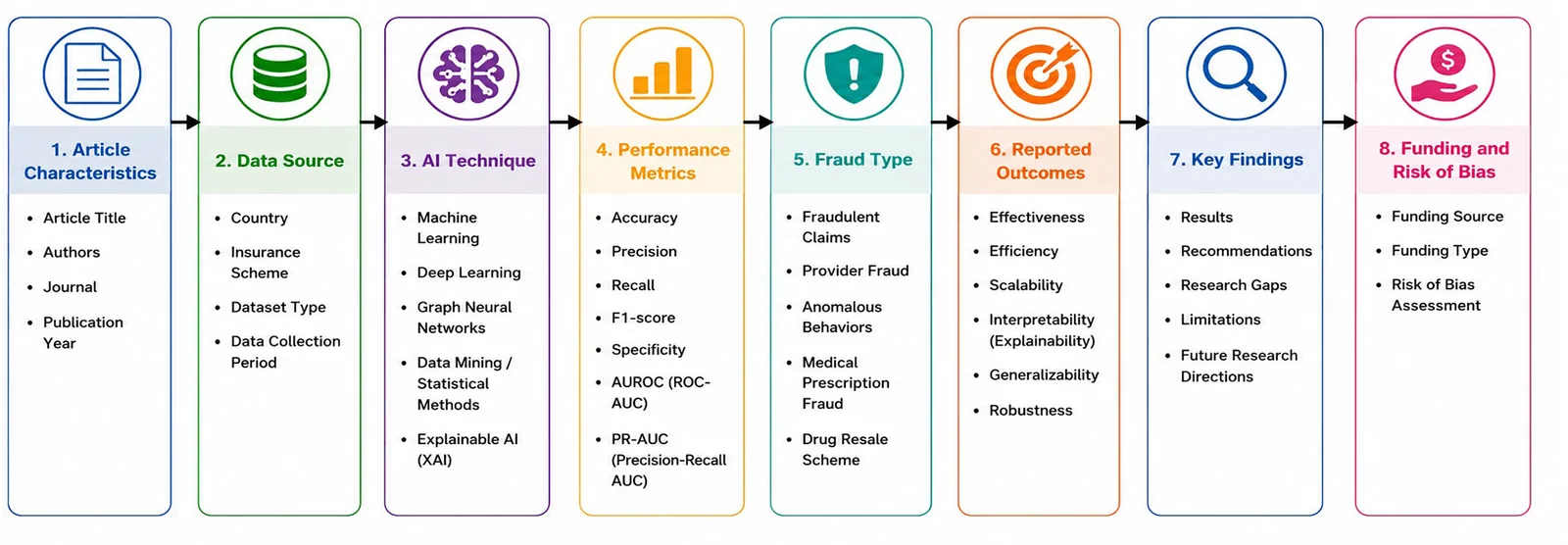
Conceptual framework illustrating the standardized data extraction domains used for the synthesis of studies included in the systematic review.

The extracted information was subsequently synthesized to identify methodological trends, AI approaches, fraud types, performance evaluation metrics, implementation challenges, funding patterns, and research gaps across the included studies.

### 2.5 Eligibility framework (PICO)

The eligibility framework was structured using the PICO approach (Population, Intervention, Comparison, and Outcomes) to define the review scope and establish consistent inclusion criteria (Table 2).

**Table 2:** PICO.

P–population	Healthcare providers, insurance providers, funded schemes, healthcare consumers, and medical professionals
I–intervention	AI–based approaches for health insurance fraud detection
C–comparison	Non-AI fraud detection approaches, where applicable
O–outcomes	Operational performance: accuracy, effectiveness, turnaround time Financial outcomes: efficiency, cost saving Implementation outcomes: generalizability, economic viability, scalability, quality of care

### 2.6 Study selection

The Silvi AI application was used to facilitate duplicate removal and the initial screening of titles and abstracts (50). Eligibility was assessed using the pre-defined inclusion and exclusion criteria. To improve consistency and minimize selection bias, all records that met the eligibility criteria or were considered potentially relevant were re-evaluated in a second screening round before final inclusion decisions were made. The overall selection process followed the PRISMA 2020 framework and is illustrated in Figure 2.

**Figure 2:**
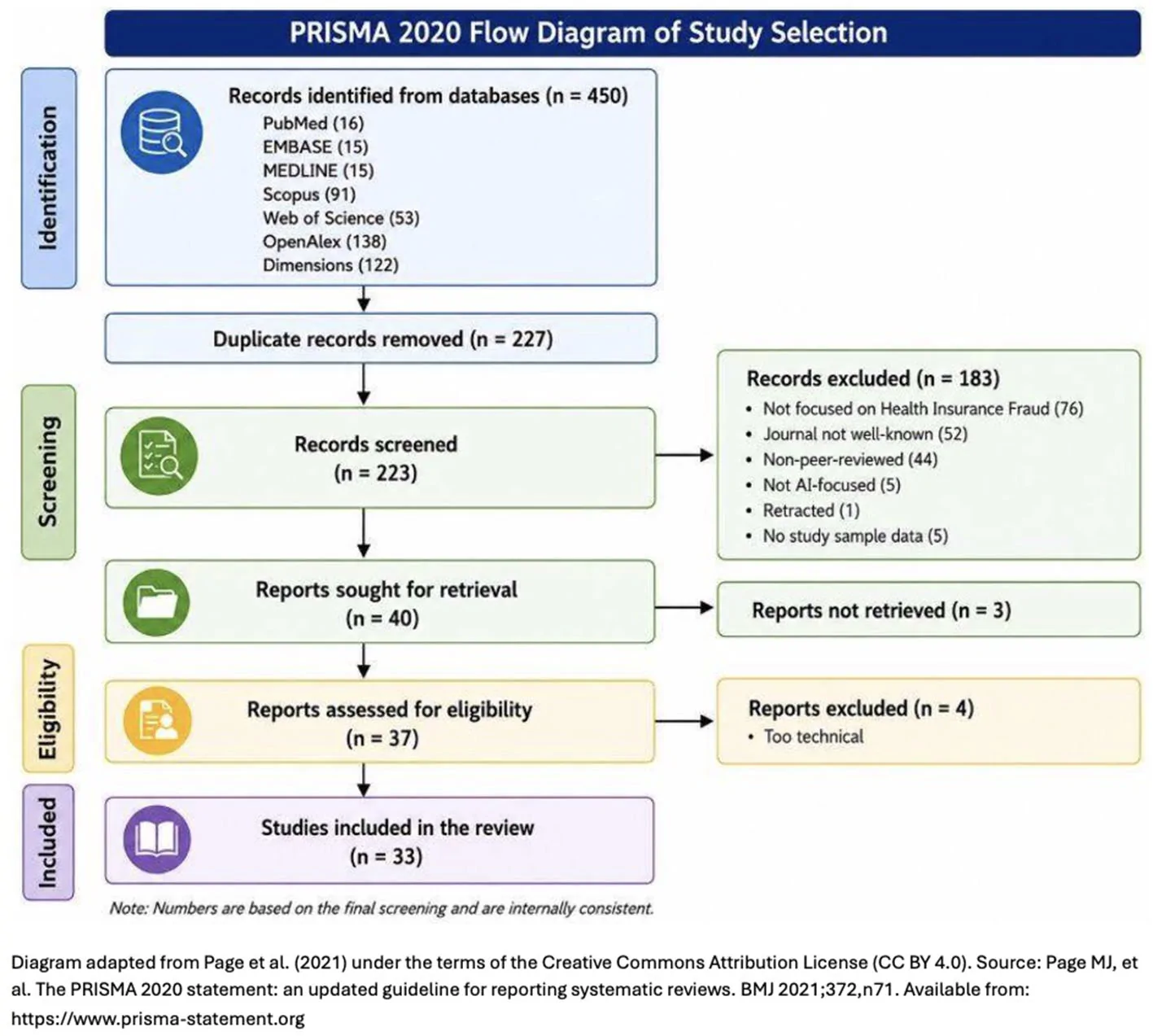
PRISMA 2020 flow diagram illustrating the study identification, screening, eligibility assessment, and inclusion process for the systematic review of artificial intelligence applications in health insurance fraud detection.

A total of 450 records were identified through database searching. After removing 227 duplicate records, 223 studies remained for title and abstract screening. Of these, 183 records were excluded according to the pre-defined eligibility criteria, and 40 reports were sought for retrieval. Three reports could not be retrieved, leaving 37 full-text articles for eligibility assessment. Four studies were subsequently excluded for not meeting the inclusion criteria, leaving a final sample of 33 studies in the systematic review. The included studies were synthesized across eight pre-defined domains: article characteristics, data source, AI technique, fraud type, performance metrics, reported effectiveness, key findings, funding, and study limitations.

### 2.7 Methodological quality assessment

The methodological quality of the included studies was assessed using the Drummond 10-item checklist, a widely recognized framework for evaluating methodological quality in health-related intervention and economic evaluation studies (47). The checklist evaluates 10 methodological domains, including the clarity of the research question, description of methodological alternatives, assessment of effectiveness, identification and measurement of costs and consequences, valuation of outcomes, adjustment for differential timing, incremental analysis, treatment of uncertainty, and completeness of reporting.

The quality assessment was conducted by the corresponding author after completion of the study selection process. To enhance consistency and reliability, each included study was assessed twice at separate time points using the same standardized evaluation criteria, and any discrepancies identified during reassessment were resolved through careful re-examination of the original publication. Each study was subsequently classified according to its overall methodological quality based on its level of compliance with the Drummond checklist.

As presented in Table 3, 31 of the 33 included studies were rated as Good, indicating strong methodological quality across most assessment domains. Many studies clearly defined their research objectives, described methodological alternatives, and demonstrated the effectiveness of their proposed AI-based fraud detection approaches (4, 10, 21). However, fewer studies adequately reported economic evaluation components, including implementation costs, operational efficiency, cost-effectiveness, and financial sustainability (7, 22–24). Comprehensive economic evaluation, including cost measurement, uncertainty analysis, and long-term implementation costs, was generally limited across the included studies. Consequently, although the overall methodological quality of the included studies was high, future research would benefit from more comprehensive reporting of implementation costs, economic outcomes, external validation, and real-world deployment considerations.

**Table 3:** Quality assessment using the Drummond checklist.

Study	Is the research question well-defined?	Comprehensive description of alternatives?	Is the effectiveness of the program established?	Are the essential and relevant costs and consequences for each alternative identified?	Costs and consequen-ces measured accurately and appropriat-ely?	Costs and conseque-nces valued credibly?	Costs and consequ-ences adjusted for differential timing?	Incremental analysis of costs and consequen-ces performed?	Allowance made for uncer-tainty in estima-tes?	Presentation and discussion of study results include all issues of concern to users?	Score
Amponsah (22)	✓	✓	✓	✓	✓	✓	✓	✓	✓	✓	Good
Bauder and Khoshgoftaar (10)	✓	✓	✓	✓	✓	✓	x	✓	✓	✓	Good
Chen et al. (28)	✓	✓	✓	✓	✓	✓	✓	✓	x	✓	Good
De Meulemeester et al. (7)	✓	✓	✓	✓	x	✓	x	✓	x	✓	Average
Devaguptam et al. (23)	✓	✓	✓	✓	✓	✓	✓	✓	✓	✓	Good
Fursov et al. (31)	✓	✓	✓	✓	✓	✓	✓	✓	✓	✓	Good
Gao et al. (37)	✓	✓	✓	✓	✓	✓	✓	✓	✓	✓	Good
Guo et al. (38)	✓	✓	✓	✓	✓	✓	✓	✓	✓	✓	Good
Haddad et al. (27)	✓	✓	✓	✓	✓	✓	✓	✓	✓	✓	Good
Hamid et al. (39)	✓	✓	✓	✓	✓	✓	✓	✓	✓	✓	Good
Hancock et al. (4)	✓	✓	✓	✓	✓	✓	x	✓	x	✓	Good
He et al. (40)	✓	✓	✓	✓	✓	✓	✓	✓	x	✓	Good
Herland et al. (2)	✓	✓	✓	✓	✓	✓	x	✓	✓	✓	Good
Hong et al. (6)	✓	✓	✓	✓	✓	✓	✓	✓	✓	✓	Good
Johnson and Nagarur (41)	✓	✓	✓	✓	✓	✓	x	✓	x	✓	Good
Johnson and Khoshgoftaar (3)	✓	✓	✓	✓	✓	✓	✓	✓	✓	✓	Good
Kaddi and Patil (11)	✓	✓	✓	✓	✓	✓	x	✓	x	✓	Good
Kurniadi et al. (12)	✓	✓	✓	✓	✓	✓	x	✓	x	✓	Good
Li (24)	✓	✓	✓	✓	x	✓	✓	✓	✓	✓	Good
Lu et al. (29)	✓	✓	✓	✓	✓	✓	x	✓	x	✓	Good
Mambo and Kimwele (42)	✓	✓	✓	✓	✓	✓	✓	✓	x	✓	Good
Mardani et al. (30)	✓	✓	✓	✓	✓	✓	✓	✓	✓	✓	Good
Mary and Claret (33)	✓	✓	✓	✓	✓	✓	✓	✓	x	✓	Good
Matloob et al. (26)	✓	✓	✓	✓	✓	✓	✓	✓	✓	✓	Good
Nabrawi and Alanazi (5)	✓	✓	✓	✓	✓	✓	✓	✓	✓	✓	Good
Rehman et al. (32)	✓	✓	✓	✓	✓	✓	✓	✓	✓	✓	Good
Sakil et al. (36)	✓	✓	✓	✓	✓	✓	✓	✓	✓	✓	Good
Salau et al. (25)	✓	✓	✓	✓	✓	✓	✓	✓	✓	✓	Good
Settipalli and Rao (43)	✓	✓	✓	✓	✓	✓	✓	✓	✓	✓	Good
Smita et al. (34)	✓	✓	✓	✓	✓	✓	✓	✓	✓	✓	Good
Sowah and Yankson (44)	✓	✓	✓	✓	✓	✓	✓	✓	✓	✓	Good
Sumalatha and Reddy (45)	✓	✓	✓	✓	x	✓	x	✓	x	✓	Average
Yoo et al. (21)	✓	✓	✓	✓	✓	✓	✓	✓	✓	✓	Good
Zhou et al. (35)	✓	✓	✓	✓	✓	✓	✓	✓	x	✓	Good

### 2.8 Data synthesis

The included studies demonstrated substantial heterogeneity in publication year, geographic location, data source, AI methodology, and evaluation practices. As shown in Figure 3, publication output and citation frequency increased markedly after 2021, reflecting growing research interest in AI-based health insurance fraud detection. Research activity was geographically concentrated, with China contributing the largest number of studies, followed by the United States and India (Figure 4). A similar pattern was observed for research funding (Figure 5), where most funded studies originated from China. This concentration reflects differences in national research investment, healthcare infrastructure, and access to large-scale insurance datasets. Consequently, many AI models were developed and validated using country-specific reimbursement systems, coding practices, and fraud characteristics, which may limit the external validity and generalizability of these findings to other healthcare systems. Broader international collaboration and external validation across diverse healthcare environments are therefore needed to improve the global applicability of AI-based fraud detection models.

**Figure 3:**
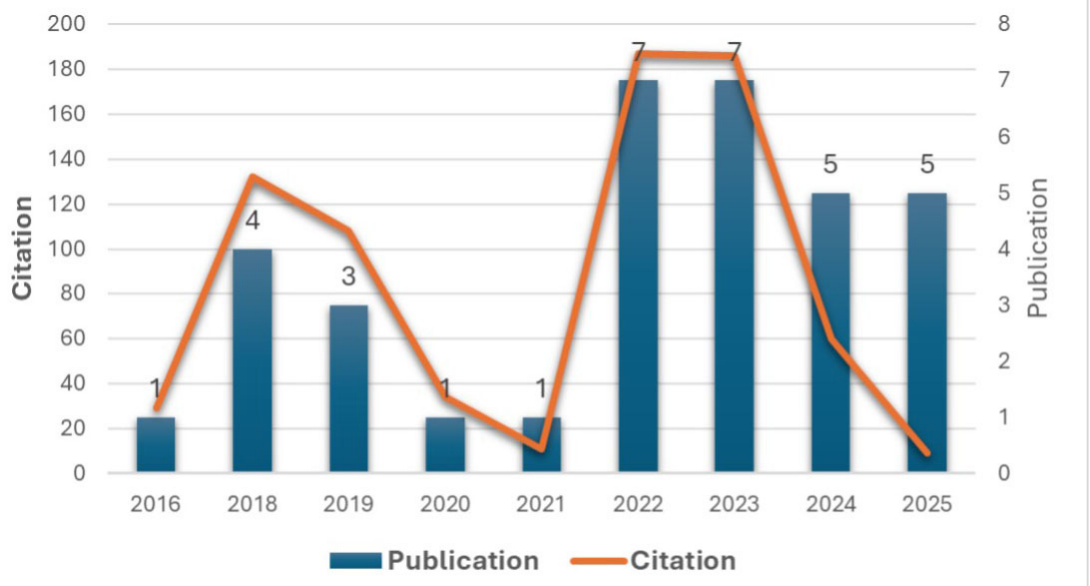
Annual publication output and citation trends of the included studies on artificial intelligence in health insurance fraud detection (2016–2025).

**Figure 4:**
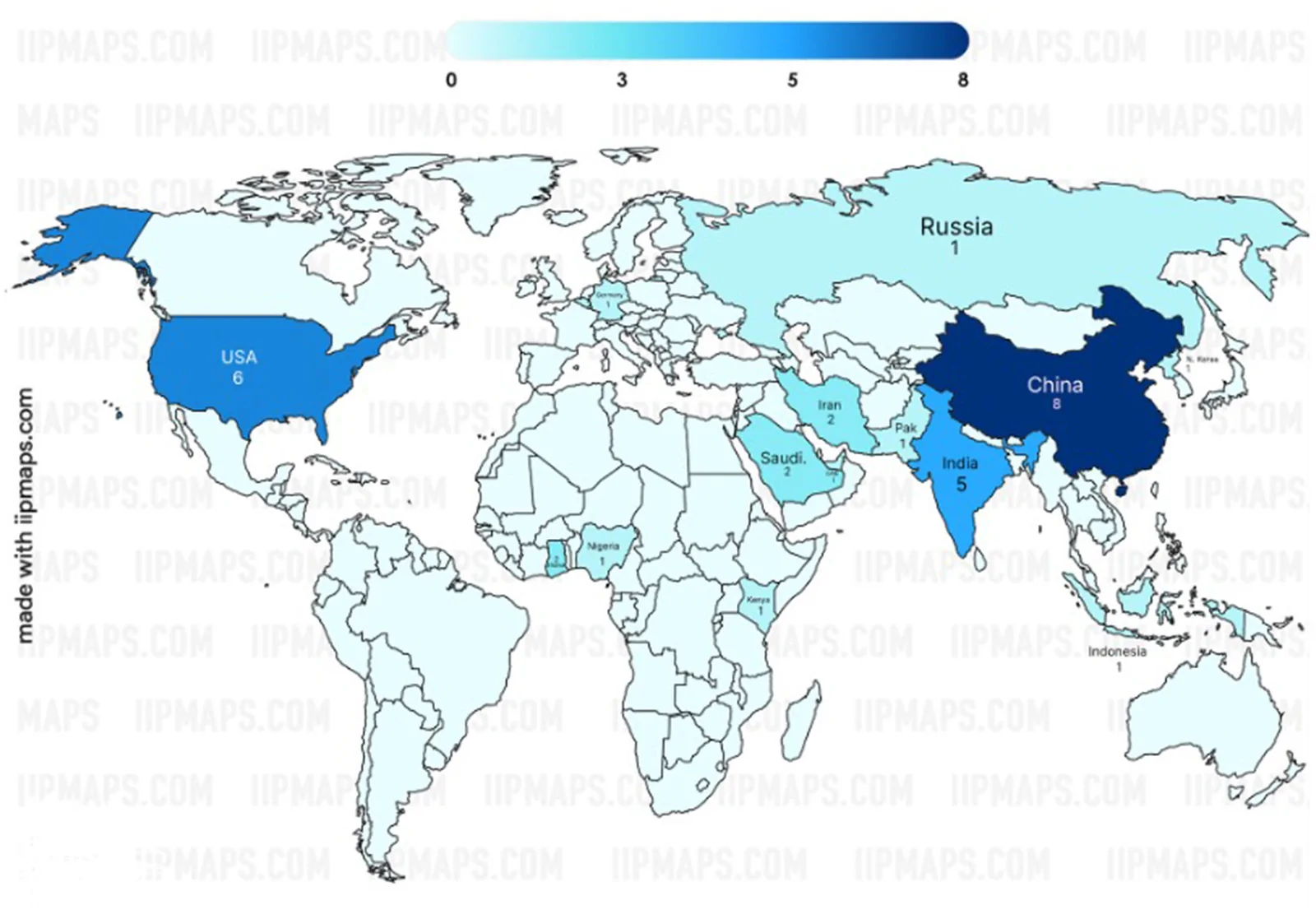
Geographic distribution of the included studies by country, illustrating the global research landscape of artificial intelligence applications in health insurance fraud detection. One study involved collaboration between two countries.

**Figure 5:**
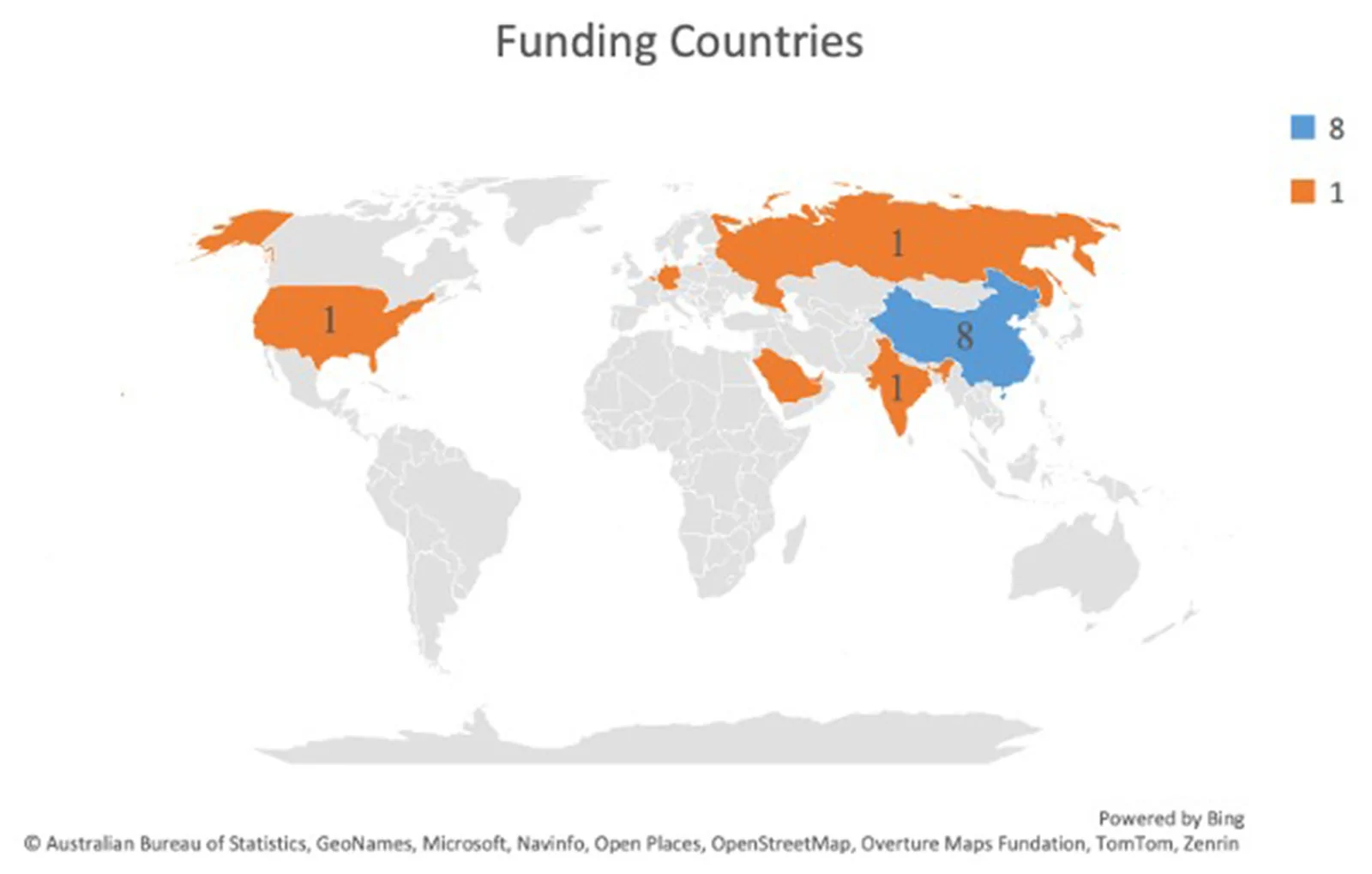
Distribution of funded studies by country among the included publications, highlighting the concentration of research funding across geographic regions.

The data sources and reported fraud manifestations varied considerably across the 33 included studies (Table 4). To improve conceptual consistency, the fraud categories were reviewed and organized according to the health insurance fraud manifestation framework described by Villegas-Ortega et al. (15). This approach classifies fraud according to specific manifestations rather than broad or overlapping categories, thereby facilitating more meaningful comparison across the included studies. Municipal and international health insurance schemes were equally represented (*n* = 6 each). Only one study employed a simulated dataset, indicating a preference for real-world claims data. The type of health insurance fraud also varied, with fraudulent claims leading in this category (*n* = 24). More specialized fraud types, such as medical prescription fraud and drug resale schemes were each addressed by only one study, indicating limited exploration of these areas.

**Table 4:** Summary of data sources and fraud types.

Study characteristic	Category	Studies
Data source	Open-source datasets (e.g., Medicare Part B)	9^*^
	National scheme	2
	Municipal scheme	6
	International scheme	
	Simulated dataset	1
Fraud type	Fraudulent claims	24
	Provider fraud	6
	Anomalous behaviors	1
	Medical prescription fraud	
	Drug resale scheme	

^*^One study utilized two open-source datasets.

Figure 6 presents the VOSviewer keyword co-occurrence network, illustrating the major research themes in AI-based health insurance fraud detection. The network highlights artificial intelligence, machine learning, fraud detection, healthcare claims, and data mining as the dominant research areas, reflecting the increasing adoption of AI for detecting fraudulent healthcare claims. Emerging topics such as graph neural networks and explainable AI, indicate a shift toward more advanced and interpretable AI approaches. However, implementation-related themes, including governance, economic evaluation, and real-world deployment, remain comparatively underrepresented, suggesting important directions for future research.

**Figure 6:**
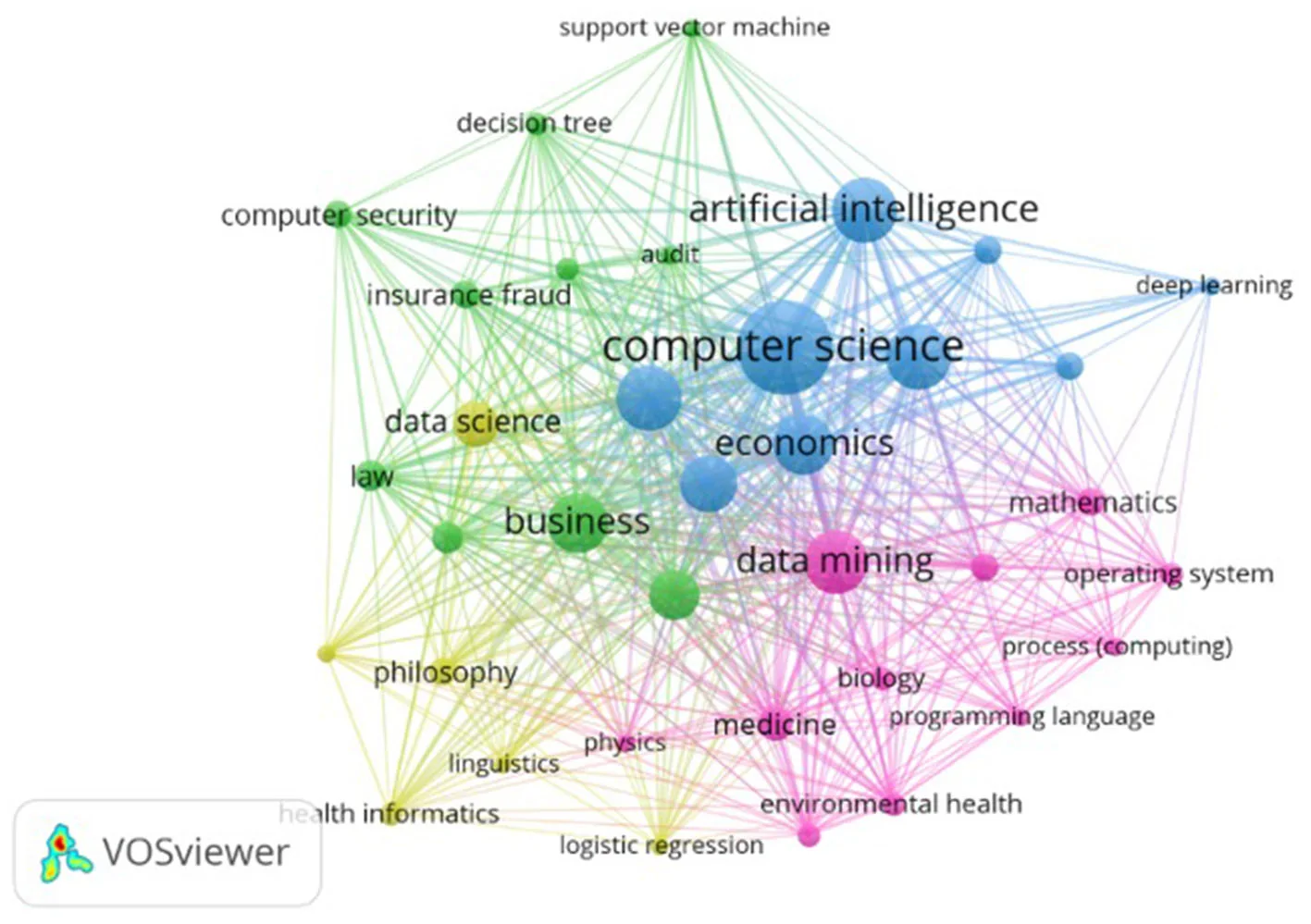
VOSviewer keyword co-occurrence network of the included studies, illustrating the relationships among major research themes in artificial intelligence-based health insurance fraud detection.

Table 5 summarizes the AI approaches reported across the included studies using a consistent methodological classification. Machine learning was the predominant category, followed by deep learning, graph-based learning, data mining, and unsupervised learning approaches. Several studies employed hybrid frameworks that combined multiple AI techniques, including deep learning, graph neural networks, explainable AI, and blockchain-enabled architectures. Because many studies implemented more than one AI technique, the total frequency exceeds the number of studies included. The findings demonstrate an increasing shift from traditional machine learning algorithms toward more sophisticated hybrid and graph-based AI approaches capable of modeling complex fraud relationships while improving detection performance.

**Table 5:** Classification of AI approaches reported in the included studies (*n* = 33).

AI category	Representative methods	No. of studies
Machine learning	Logistic Regression, Decision Tree, Random Forest, Support Vector Machine (SVM), K-Nearest Neighbors (KNN), XGBoost	30
Deep learning	Artificial Neural Networks (ANN), Convolutional Neural Networks (CNN), Deep Neural Networks (DNN)	6
Graph-based learning	Graph Neural Networks (GNN), Graph Attention Networks (GAT), Heterogeneous Graph Models	4
Unsupervised learning	Anomaly Detection, Manifold Learning, Bi-clustering	5
Data mining	Association Rule Mining, Multinomial Naïve Bayes, Pattern Mining	5
Hybrid AI frameworks	Machine Learning + Blockchain, CNN–Transformer–XGBoost, Multi-stage AI Frameworks	5

Several studies employed more than one AI approach; therefore, a single study may contribute to multiple categories. Consequently, the total frequency exceeds the number of included studies (n = 33).

### 2.9 Risk of bias and limitations

The criteria to examine the risk of bias included the availability of funding, as well as study limitations claimed by the authors, as shown in Table 6. The potential risk of bias is not due to the country of origin of the research, but to a situation where one or more of the authors is affiliated with the funder or institution that sponsored the research. Such affiliations could create a conflict of interest in study design, data interpretation, or reporting. As such, authors' affiliation with the funder was regarded as a potential for bias during our assessment, as is done in other risk of bias and conflict of interest evaluation frameworks.

**Table 6:** Limitations.

Limitations	Studies
Size, access, and quality of datasets	19
Changes in fraudulent behavior	2
Limited interpretability	1
Cost ramifications	1

Some studies reported more than one limitation.

The most frequently cited limitations related to the size, access, and quality of datasets (*n* = 19), reflecting challenges in obtaining comprehensive health insurance data. Fewer studies reported limitations related to changing fraudulent behaviors (*n* = 2), or cost ramifications (*n* = 1).

No authors reported conflicts of interest, and 16 studies were funded, with most originating from China (*n* = 8). The remaining funded studies were from the United States, Belgium, India, Russia, Germany, the United Arab Emirates, Saudi Arabia, and Korea (one each), with one study receiving multiple funding sources. Potential biases include reliance on open datasets, limited regional representation, particularly in UAE (*n* = 1) and Saudi Arabia (*n* = 2), and author affiliation with funder in some studies out of China.

## 3 Results of individual studies

The performance metrics reported across the included studies varied considerably, reflecting substantial heterogeneity in evaluation practices (Table 7). Recall (*n* = 18), Accuracy (*n* = 17), Precision (*n* = 16), F1-score (*n* = 15), and AUROC (*n* = 13) were the most frequently reported evaluation metrics. However, because health insurance fraud datasets are inherently highly imbalanced, with fraudulent claims representing only a small proportion of the total observations, accuracy alone provides an incomplete assessment of classifier performance. Several studies explicitly addressed class imbalance using data-level techniques such as random under-sampling, the Synthetic Minority Over-sampling Technique (SMOTE), and SMOTE-ENN, as well as algorithm-level approaches including ensemble learning, graph-based learning, and cost-sensitive classification (10–13). Consequently, many studies emphasized Recall, F1-score, AUROC, and PR-AUC as more appropriate performance measures because they provide a more balanced evaluation of fraud detection models by accounting for the correct identification of minority fraud cases while reducing the influence of the dominant non-fraud class. Overall, the findings demonstrate increasing recognition of the importance of adopting robust evaluation metrics and class-imbalance handling techniques to improve the reliability and practical applicability of AI-based health insurance fraud detection systems.

**Table 7:** Performance metrics reported in the included studies.

Performance metric	No. of studies
Recall	18
Accuracy	17
Precision	16
F1-score	15
AUROC (area under the receiver operating characteristic curve)	13
Specificity	4
PR-AUC (area under the precision–recall curve)	3
Confusion matrix	2
Positive likelihood ratio (LR+)/negative likelihood ratio (LR–)	1
Cohen's Kappa statistic	1
G-measure (G-mean)	1

Several studies reported more than one performance metric; therefore, the total frequency exceeds the number of included studies (*n* = 33).

Table 8 presents the accuracy values reported by some studies as a performance metric. Reported accuracy ranged from 75 to 99.4%, reflecting substantial variability. Several studies reported accuracy values above 95%, while others demonstrated moderate performance. One study did not report accuracy, although the used fraud detection methods were claimed to improve accuracy. Given the highly imbalanced nature of datasets, accuracy values should be interpreted alongside complementary metrics such as recall and AUROC.

**Table 8:** Best-performing algorithms and reported accuracy in included studies.

Study	Best-performing algorithm/model	Reported accuracy
Amponsah (22)	Machine learning–Blockchain framework	98.00%
Bauder and Khoshgoftaar (10)	Random forest (RF)	97.96%
Devaguptam et al. (23)	Hybrid AI-based fraud detection framework	94.67%
Haddad et al. (27)	Unsupervised data mining algorithm	75.00%
He et al. (40)	Intelligent supervision framework	87.00%
Herland et al. (2)	Logistic regression with improvement strategy	87.40%
Hong et al. (6)	Multi-channel heterogeneous graph neural network	**NR**
Johnson and Nagarur (41)	Multi-stage fraud detection framework	87.00%
Kaddi and Patil (11)	Ensemble learning model	91.50%
Kurniadi et al. (12)	XGBoost with instance hardness thresholding	98.41%
Mary and Claret (33)	Big data-based machine learning model	96.78%
Matloob et al. (26)	Adaptive deep learning model	97.49%
Nabrawi and Alanazi (5)	XGBoost	98.21%
Rehman et al. (32)	Deep learning framework	97.40%
Sakil et al. (36)	CNN–Transformer–XGBoost	94.00%
Salau et al. (25)	Deep learning model	99.40%
Sowah and Yankson (44)	Genetic support vector machine (GSVM)	87.91%

The reported accuracy corresponds to the best-performing algorithm or framework reported in each study. Several studies evaluated multiple machine learning models; therefore, only the model associated with the reported accuracy is presented. Accuracy values should be interpreted alongside complementary evaluation metrics such as Precision, Recall, F1-score, PR-AUC, and AUROC (see Table 8), particularly because health insurance fraud detection datasets are typically highly imbalanced. NR, not reported. The original study did not report accuracy as an evaluation metric.

In addition, Table 8 summarizes the best-performing algorithms together with the corresponding reported accuracy values. Accuracy is presented for comparison purposes only because not all studies report the same evaluation metrics. Complementary performance measures, including Precision, Recall, F1-score, AUROC, and PR-AUC, are summarized in Table 8 to provide a more comprehensive evaluation of AI-based fraud detection performance.

### 3.1 Common characteristics of the included studies

Table 9 summarizes common characteristics across the included studies. All studies provided recommendations (*n* = 33), and the majority reported superiority over existing methods (*n* = 30), highlighting the effectiveness of AI-based approaches. Efficiency-related outcomes were discussed in nearly half of the studies (*n* = 16), while cost-effectiveness (*n* = 9) and runtime efficiency (*n* = 8) were less frequently addressed. Only a small number of studies discussed generalizability or limited interpretability. Most studies acknowledged limitations (*n* = 22), indicating methodological and implementation challenges.

**Table 9:** Common characteristic of the included studies.

Characteristic	Studies
Recommendations	33
Improved performance	30
Limitations	22
Efficiency	16
Cost effectiveness	9
Runtime efficiency	8
Generalizability	3
Limited interpretability	2

## 4 Findings and discussion

### 4.1 Effectiveness of AI-based approaches

All the studies included established the effectiveness of AI-based methods using performance metrics. Machine learning and deep learning approaches consistently demonstrated superior performance compared to traditional statistical techniques (3, 25, 26). Reported accuracy values ranged from 75% to over 99% depending on the dataset and modeling approach employed (12, 27).

Graph-based and heterogeneous network approaches showed promise in detecting collusive fraud schemes, outperforming conventional classifiers (28–30, (51)). Similarly, ensemble learning and hybrid frameworks demonstrated robustness in highly imbalanced datasets (4, 11).

### 4.2 Incremental value and comparative performance

Most included studies compared their proposed AI models with conventional machine learning or statistical approaches to evaluate incremental improvements in fraud detection performance. Across the reviewed literature, improvements were most frequently demonstrated using Recall and AUROC, which were reported by 18 and 13 studies, respectively (Table 8). These metrics are particularly valuable for highly imbalanced health insurance fraud datasets because they provide a more reliable assessment of a model's ability to identify minority fraud cases than overall accuracy alone.

Deep learning and graph-based approaches demonstrated notable advantages over conventional machine learning algorithms. Deep learning models were able to learn complex, non-linear relationships within healthcare claims data, particularly in studies where feature engineering, graph representations, or other data transformation techniques increased the dimensionality of the input data, thereby improving the identification of subtle fraud patterns. However, the dimensionality of healthcare claims datasets varied considerably across the included studies and depended on the characteristics of the original datasets as well as the extent of feature engineering and data preprocessing performed before model development. Similarly, graph neural networks and heterogeneous graph models explicitly represented relationships among healthcare providers, patients, medical institutions, and insurance claims, enabling the detection of organized fraud networks and collusive behaviors that are difficult to identify using traditional feature-based classifiers (6, 21, 31, 32). These capabilities explain why graph-based methods generally achieved higher Recall and AUROC values when detecting complex fraud scenarios.

Although AI-based approaches consistently demonstrated improved predictive performance compared with conventional techniques, the reported improvements were primarily evaluated using technical performance metrics. Relatively few studies assessed operational feasibility, computational efficiency, implementation costs, or scalability within real-world healthcare environments (7, 24). Likewise, only a limited number of investigations considered economic outcomes or implementation challenges associated with deploying AI-based fraud detection systems in routine healthcare practice (22, 23). Consequently, while the available evidence indicates that AI-based methods provide measurable improvements in fraud detection accuracy and robustness, further research is required to evaluate their cost-effectiveness, external validity, and practical implementation across diverse health insurance systems.

### 4.3 Reporting interpretability, and practical implications

Most studies demonstrated strong reporting practices (33–35). However, interpretability was explicitly addressed in only a small subset of studies, despite its regulatory importance (7, 36). Broader implementation considerations such as governance and regulatory compliance were rarely examined. This gap highlights a continued emphasis on technical performance over policy relevance. Overall, the reviewed studies demonstrate that advanced machine learning and data mining approaches can substantially improve health insurance fraud detection, particularly when applied to large and complex healthcare claims datasets. Nevertheless, technical performance alone is insufficient for successful implementation. Broader adoption of AI-based fraud detection systems will require improved model interpretability, standardized evaluation frameworks, external validation across diverse healthcare settings, and greater attention to governance, privacy, and operational feasibility.

### 4.4 Research gaps, limitations, and future directions

Despite the encouraging performance of artificial intelligence (AI) techniques in health insurance fraud detection, several methodological and practical limitations remain. First, considerable heterogeneity was observed across the included studies with respect to datasets, fraud definitions, AI methodologies, and performance evaluation metrics. Although accuracy, precision, and recall were frequently reported, relatively few studies employed more robust evaluation measures such as the F1-score, PR-AUC, or Matthews Correlation Coefficient, which are generally considered more appropriate for highly imbalanced healthcare fraud datasets. This lack of standardized reporting limits meaningful comparisons across studies and complicates the identification of superior fraud detection approaches.

A second limitation concerns dataset availability and representativeness. Most studies relied on publicly available datasets, Medicare claims, or country-specific health insurance databases, with relatively limited evidence from low- and middle-income countries. Restricted access to real-world insurance data, driven by privacy regulations and commercial confidentiality, remains a major barrier to developing and validating robust AI models. Consequently, the external validity and generalizability of many proposed approaches remain uncertain when applied to different healthcare systems.

The review also identified methodological gaps in the implementation of AI models. While machine learning, deep learning, graph neural networks, and ensemble methods consistently demonstrated promising predictive performance, relatively few studies investigated model interpretability, fairness, transparency, or regulatory compliance. Explainable AI techniques were incorporated in only a limited number of studies despite their importance for supporting decision-making by insurers, healthcare providers, and policymakers. Furthermore, economic evaluations, implementation costs, computational efficiency, and real-world deployment strategies were infrequently reported, limiting the practical translation of research findings into operational fraud detection systems.

Another important finding is the inconsistent treatment of class imbalance, which is an inherent characteristic of health insurance fraud datasets. Although several recent studies employed sampling techniques such as SMOTE, SMOTE-ENN, undersampling, or ensemble learning to improve fraud detection performance, no standardized methodological framework has emerged.

Based on the evidence synthesized in this review, future research should prioritize the development of standardized benchmark datasets, transparent reporting guidelines, explainable and trustworthy AI models, privacy-preserving learning approaches such as federated learning, and prospective validation using real-world health insurance data. Greater attention should also be given to interdisciplinary collaboration among computer scientists, healthcare professionals, insurance organizations, and policymakers to ensure that AI-enabled fraud detection systems are accurate, interpretable, economically feasible, and suitable for large-scale implementation across diverse healthcare environments.

### 4.5 Responsible implementation of AI-based health insurance fraud detection systems

The evidence synthesized in this review indicates that successful implementation of AI-based health insurance fraud detection depends not only on predictive performance but also on appropriate organizational, technical, and regulatory practices. Based on the findings of the included studies, several recommendations can be proposed to support the responsible implementation of AI-enabled fraud detection systems.

First, AI models should complement rather than replace expert investigators. Human oversight remains essential for reviewing high-risk claims, validating model outputs, and minimizing false-positive decisions that may adversely affect healthcare providers or beneficiaries. Second, healthcare organizations should adopt explainable AI techniques wherever possible to improve transparency and increase confidence in automated fraud detection decisions among investigators, insurers, regulators, and other stakeholders.

Third, AI systems should undergo external validation using datasets from different healthcare systems before large-scale deployment. Most studies included in this review relied on country-specific datasets, particularly from China and the United States, which may limit the generalizability of reported performance. Independent validation would improve confidence in model robustness across diverse healthcare environments.

Fourth, healthcare organizations should establish governance mechanisms for AI deployment, including clear accountability, routine monitoring of model performance, periodic model updating to address evolving fraud patterns, and compliance with applicable privacy and data protection regulations. Continuous auditing is particularly important because fraudulent behaviors continuously evolve over time.

Finally, future implementation studies should move beyond reporting predictive performance alone by evaluating operational efficiency, implementation costs, organizational readiness, user acceptance, and cost-effectiveness under real-world conditions. Such evidence will support informed decisions regarding the adoption and long-term sustainability of AI-enabled fraud detection systems.

Collectively, these recommendations provide a practical framework for translating advances in artificial intelligence into trustworthy, transparent, and sustainable health insurance fraud detection systems while maintaining fairness, accountability, and regulatory compliance.

## 5 Conclusion

This systematic review synthesized the global evidence on the application of artificial intelligence (AI) in health insurance fraud detection using a PRISMA-guided methodology. The findings demonstrate that AI has become an increasingly important approach for detecting fraudulent healthcare claims, with machine learning remaining the most widely adopted methodology, followed by deep learning, graph-based learning, and hybrid AI frameworks. Across the included studies, AI-based models generally outperformed conventional fraud detection approaches, particularly when evaluated using performance measures such as Recall, F1-score, and AUROC, which are better suited to highly imbalanced healthcare fraud datasets.

Despite these advances, the review identified considerable heterogeneity in datasets, fraud types, AI methodologies, evaluation metrics, and reporting practices. Most studies relied on country-specific datasets, particularly from China and the United States, limiting the generalizability of their findings to other healthcare systems. Furthermore, relatively few investigations addressed model interpretability, external validation, economic evaluation, implementation costs, governance, or regulatory considerations. The lack of standardized reporting and consistent performance evaluation also limits meaningful comparison across studies.

This review has several limitations. The literature search was restricted to peer-reviewed English-language publications published between 2016 and 2025 and included only studies retrieved through the pre-defined search strategy and eligibility criteria. Consequently, relevant studies published outside the search period, indexed after the search was completed, or reported in other languages may not have been captured. In addition, the methodological heterogeneity of the included studies precluded quantitative meta-analysis.

Future research should focus on developing standardized benchmark datasets, improving the reporting of evaluation metrics for imbalanced datasets, strengthening external validation across diverse healthcare systems, and integrating explainable and trustworthy AI into fraud detection frameworks. Greater emphasis should also be placed on privacy-preserving learning, fairness, economic evaluation, and real-world implementation to facilitate the responsible and scalable deployment of AI-enabled health insurance fraud detection systems. Future research should not only focus on improving AI algorithms but also on generating evidence to support their responsible implementation in routine health insurance practice through standardized evaluation, external validation, governance frameworks, and real-world implementation studies.

## Data Availability

The original contributions presented in the study are included in the article/supplementary material, further inquiries can be directed to the corresponding author.
